# Profiles of Stress and Coping Associated With Mental, Behavioral, and Internet Use Problems Among Adolescents During the COVID-19 Pandemic: A Stratified Random Sampling and Cluster Analysis

**DOI:** 10.3389/fpubh.2022.826911

**Published:** 2022-03-29

**Authors:** Rui She, Keiman Wong, Jiaxi Lin, Youmin Zhang, Kinlong Leung, Xue Yang

**Affiliations:** Center for Health Behaviours Research, The Jockey Club School of Public Health and Primary Care, Faculty of Medicine, The Chinese University of Hong Kong, Hong Kong, Hong Kong SAR, China

**Keywords:** stress-coping theory, mental health, behavioral problems, Internet use, youth, cluster analysis

## Abstract

**Background:**

Adolescents are vulnerable to behavioral and mental health problems, which might be further exacerbated during the COVID-19 pandemic. This study explored how participants with different profiles of stressful life events, coping resources (i.e., self-esteem and perceived social support) and coping strategies (i.e., maladaptive and adaptive coping) varied in the prevalence of mental, behavioral, and Internet use problems.

**Methods:**

Data were collected from a large and representative sample of Chinese secondary school students in Hong Kong (*n* = 3,136) from September to November 2020 (48.1% males; mean age = 13.6 years old). Cluster analysis and logistic regression models were used for analysis.

**Results:**

The prevalence of suicidal ideation and sleep disturbance was 29.8 and 55.4%, respectively. Behavioral problems were most frequently reported in excessive social media use (53.5%), followed by excessive Internet gaming (43.6%), obesity (34.1%), damaging properties (14.6%), and alcohol or substance abuse (5.1%). The results of cluster analysis yielded three distinctive stress and coping profiles: severe profile (High Risk/Low Protective; 17.0%), moderate profile (Moderate Risk/Moderate Protective; 35.8%), and mild profile (Low Risk/High Protective; 47.2%). Participants with severe and moderate profiles displayed significantly higher levels of mental (range for AOR: 2.08–15.06; all *p* < 0.001) and behavioral health problems (range for AOR: 1.22–11.22; all *p* < 0.05) compared to the mild profile cluster.

**Conclusions:**

Adolescents' mental and behavioral health may be shaped by a combination of stressful life events and variations in coping resources as well as strategies. Transdiagnostic and multimodal interventions on these factors are warranted to reduce mental, behavioral, and Internet use problems among adolescents.

## Introduction

Adolescence is a period of stressful challenges, during which individuals undergo extensive physical, psychological, emotional, and personality development. The high prevalence and co-occurrence of behavioral and mental health problems in adolescence have emerged as a paramount public health concern. For instance, mental disorders affect 10–20% of youth worldwide; and suicide is the third leading cause of death in 15–19 year-old youth (1). A national survey in the U.S. reported that 7.4% of children aged 3–17 years had a current behavioral problem (2). More importantly, the coronavirus 2019 (COVID-19) pandemic poses a grim challenge to youths' normal life, including lockdowns and schooling disruptions (3). Adolescents are particularly vulnerable to mental distress and behavioral difficulties in pandemics (4). A systematic review reported that 41.7, 42.3, 30.8, and 21.3% of children and adolescents suffered from depression, irritability, inattention, and sleep disturbance during the COVID-19 pandemic, respectively (4). Internet-related behaviors (e.g., Internet gaming) also had increased during the pandemic among Chinese adolescents due to social distancing and motivation of escapism coping (5, 6). Therefore, it is critical to raise the awareness of monitoring various behavioral and mental health problems among adolescents during this unusual period.

Adolescents are commonly exposed to various risks and stressors related to interpersonal relationships, academic stress, violence, and health threats (7). The high levels of stressful experiences often lead to disruptions in adolescents' physical, mental, and behavioral health (8, 9). However, individuals who experience negative life stress do not necessarily develop adverse health consequences. In light of this, researchers have proposed the concept of coping, which represents the cognitive and behavioral efforts of an individual to manage the internal and external demands encountered during a specific stressful situation (10). According to the stress-coping theory, coping responses in face of adversity will greatly affect one's health outcomes. Coping strategies can be adaptive (e.g., positive reframing and acceptance) or maladaptive (e.g., rumination and self-blame). Generally, adaptive coping (in which the stressor is managed cognitively or through action) is thought to mitigate the debilitating stress effects and prevent the development of depression and risky behaviors in face of stressors (11, 12), whereas maladaptive coping (in which the stressor is ignored or repressed) has a positive association with depression, suicidal ideation, and problematic behaviors (e.g., Internet-related behaviors) (13–15). On the other hand, coping resources can aid in the coping process by facilitating coping flexibility and increasing the efficacy of adopted coping strategies (10, 16). These resources can be stable characteristics of a person's disposition (e.g., self-esteem) and social environment (e.g., social support). Social support and self-esteem may have stress-buffering effects by preventing a situation from being appraised as stressful or by providing a solution to a stressful problem, which are associated with lower risks of depression and problematic behaviors (e.g., smoking and alcohol use) among adolescents (9, 17).

Despite these specific associations between stress, coping and health outcomes, there is very limited research defining the collective relationships between stressful life events, coping resources and strategies, as well as mental and behavioral health problems among adolescents. Given that adolescents tend to use multiple coping resources and strategies to deal with stress, studies examining behavioral and psychological responses to stressors should incorporate both of these constructs and capture the multidimensionality of stress-coping processes (9). Cluster analysis is a promising method for identifying and describing subgroups of individuals along multiple dimensions of interest (e.g., stressors, coping resources, and coping strategies). Such an approach may assist health professionals to identify distinct stress-coping profiles to which individuals might belong and, subsequently, shape intervention designs to the unique dispositions and risks of the targeted group.

In the present study, we aimed (1) to investigate the prevalence of behavioral (e.g., smoking, substance abuse, damaging properties, and Internet gaming) and mental health problems (e.g., suicidal ideation and sleep disturbance) in a large-scale and representative population of adolescents during the COVID-19 pandemic; (2) to identify profiles of adolescents based on their exposures to stressful life events, coping resources (i.e., self-esteem and perceived support) and individual coping strategies; and (3) to investigate whether the subgroups of adolescents created by the cluster analysis differed in levels of behavioral and mental health problems.

## Methods

### Study Design and Participants

A school-based survey was conducted among secondary school students in Hong Kong from September to November 2020 when the spread of the virus in local clusters had been controlled and the schools were re-opened after a long time of face-to-face classes suspension since January 2020. A stratified random sampling of schools was implemented with one secondary school randomly selected and invited for each of the 18 districts in Hong Kong. As a result, 13 of the 18 schools accepted the invitation to participate in this study. All secondary 1–4 (7th−10th year of formal education) students who lived in Hong Kong and attended face-to-face classes at the investigation time of the schools were invited. Shenzhen-Hong Kong cross-boundary students who lived in Shenzhen, mainland China, and attended online courses were not invited. Secondary 5 and 6 students were not invited due to schools' concerns about their academic stress and exam pressure. In total, 4,323 students from the 13 schools were invited, and 3,147 (72.8%) returned their questionnaires.

### Procedures

Students and parents were invited and informed about the survey and its purpose with school teachers' assistance. Participants were explained that the participation was voluntary and anonymous, and rejection would not affect any right or service they would receive from the school. They were also guaranteed that only the research team can access their data. Two research assistants with a training background in psychology and at least 6 months of interviewing experience implemented the survey in classroom settings in the absence of teachers. The survey questionnaire had about 100 items which took about 15 min to complete. No incentive was given to the participants. Ethics approval was obtained from the corresponding author's affiliation (Ref No. SBRE-18-433). The written informed consent was obtained from both parents and children.

### Measures

#### Problematic Behaviors

Self-reported engagement in problematic behaviors was measured using the Problem Behavior Scale, which has been commonly used among adolescents (18, 19). The frequency of behavioral health problems, including alcohol or substance addiction, tobacco use, damaging properties, running away from home, and skipping school/absenteeism, during the past 6 months were asked. As sedentary lifestyle and obesity are significant challenges during the COVID-19 pandemic for children and adolescents (20), we assessed obesity problem using the item from the Pediatric Behavior Scale to examine whether the adolescents had been overweight or gained too much weight in the past 6 months (21). Response categories were dichotomized into having problematic behaviors (sometimes/very often) or not (none).

#### Internet Use

Two items were used to ask participants about the average time per week they spent on social media and Internet gaming during the past 6 months. As recommended by the American Academy of Pediatrics that time allotted to Internet gaming should be within 1 h per day and the total screen time should not exceed 2 h for children and teenagers (22), participants who spent >1 h on social media and Internet gaming per day were classified as excessive social media and Internet gaming users, respectively.

#### Mental Health Problems

The presence of suicidal ideation was measured using item 9 of the Patient Health Questionnaire, which asked “Over the past two weeks, how often have you thought that you would be better off dead, or of hurting yourself” (23). Responses rated on a 4-point Likert-type were dichotomized into 0 (not at all) and 1 (several days/more than half of the days/nearly every day). Such measurement has been widely used in previous studies (24). The frequency of sleep disturbance in the past 6 months was measured using a single item, with responses categorized into 1 (sometimes/very often) and 0 (none).

#### Stressful Life Events

The Adolescent Self-Rating Life Events Checklist (ASLEC) was used to measure the severity of life stress experienced during the past year (8). It lists 26 negative life events on six social-stress domains: interpersonal relationship (e.g., I argued with my classmates), academic pressure (e.g., I failed in the examination), being punished (e.g., I was criticized and punished), bereavement (e.g., A family member/close friend died), the pressure of health and adaptation, and others, which were chosen on the basis of having occurred most frequently to Chinese adolescents. Participants were first asked whether the particular event happened to them. Then they were asked to rate the perceived stressfulness of each experienced event (i.e., the extent to which the event affected the respondent's life) on a five-point Likert scale ranging from 0 (not at all) to 4 (extremely severe). If a particular event did not happen, the event was scored 0, as it did not affect the respondent's life at all. Summing scores for all events in each subscale generates a total stress intensity score for the specific domain. A higher score indicates a greater perception of stress. The Cronbach's alpha of the scale was 0.94 in the present study.

#### Perceived Social Support

Four items were used to assess perceived social support, including two items about parental support and two items for peer support (25). The items were “How much support had you received from your parents/friends when you needed to talk with someone or needed emotional support?” and “How much support had you received from your family/friends when you needed instrumental support (e.g., financial support)?” The items were rated on a 10-point scale, ranging from 0 (none) to 10 (tremendous). Higher scores denote higher levels of perceived social support. The Cronbach's alpha was 0.79 in the present study.

#### Self-Esteem

Self-esteem was measured using the abbreviated version of the Rosenberg Self-esteem Scale (RSE). The original RSE is a 10-item assessment with a four-point Likert scale format ranging from strongly disagree to strongly agree. Previous studies have used different abbreviated versions of RSE, which has shown reliability and validity across age and gender groups (26). The five positive-worded items of RSE were used in the current study. It had a Cronbach's alpha of 0.90.

#### Maladaptive and Adaptive Cognitive Coping

The short version of Cognitive Emotion Regulation Questionnaire (CERQ-short) was used to assess the set of cognitive emotion regulation strategies that individuals apply in response to stressful life events (27). The original CERQ has been well-validated in Chinese studies (28, 29), which demonstrated acceptable psychometric properties (e.g., Cronbach's alphas = 0.83–0.90 and test-retest coefficients = 0.64–0.68). The CERQ-short also showed satisfactory validity, reliability, and measurement invariance (e.g., Cronbach's alpha = 0.80 and test-retest coefficients = 0.69) among adolescents across different countries (30, 31) and had been applied in Hong Kong Chinese people (32). The CERQ-short consists of nine dimensions: (i) self-blame, (ii) other-blame, (iii) rumination, (iv) catastrophizing, (v) putting into perspective, (vi) positive refocusing, (vii) positive reappraisal, (viii) acceptance, and (ix) planning. Theoretically, the nine strategies can be grouped into adaptive coping strategies (putting into perspective, positive refocusing, positive reappraisal, acceptance, and planning) or maladaptive coping strategies (self-blame, other-blame, rumination, and catastrophizing). Items were measured on a 5-point Likert scale ranging from 1 (never) to 5 (always). The Cronbach's alphas for the maladaptive coping subscale and adaptive coping subscale were relatively low but acceptable (0.57 and 0.59) (33).

#### Background Factors

Background information, including sex, age, whether being born in Hong Kong, living arrangements, and parental education levels, were collected.

### Statistical Analysis

The SPSS 23.0 Statistics for Windows (IBM Corp. Released 2015, Armonk, NY: IBM Corp) were used for all statistical analyses. The two-step cluster analysis procedure, an exploratory method to identify natural latent groupings within a dataset of continuous (standardized) and categorical features, based on the agglomerative hierarchical clustering method and assuming a joint multinomial-normal distribution was used. Such a technique presents several advantages compared to more traditional techniques, like automatically determining the optimal number of clusters by comparing the values of a model-choice criterion across different clustering solutions [i.e., log-likelihood distance and the Schwarz Bayesian Information Criterion (BIC)] rather than on an arbitrary choice, using categorical and continuous variables simultaneously, analyzing atypical values, and being able to handle large datasets (34). Comparative studies regarded two-step cluster analysis as one of the most reliable in terms of the number of subgroups detected, classification probability of individuals to subgroups, and reproducibility of findings (35). The set of indicator variables for the clustering procedure carried out included the six subscales of stressful life events, self-esteem, family support, friend support, maladaptive coping, and adaptive coping. The clustering quality for the final model was estimated with the Silhouette index, which constitutes a global consistency measure of the cohesion/separation. The Silhouette index is a commonly used indicator to measure how tightly grouped all the data in the cluster (36). Silhouette values fall within the range of −1 to 1 and higher values denote better matching in one's own cluster (similarity within the cluster) and poor matching in other clusters (different compared to the other clusters). Values lower than 0.30 are interpreted as having a poor fit, between 0.30 and 0.50 as fair, and higher than 0.50 as good (37).

Chi-square tests and analysis of variance (ANOVA) with Fisher's least significant difference (LSD) *post-hoc* test were used to confirm whether individuals of clusters differed significantly in sociodemographic characteristics and indicators included in the cluster analysis; effect sizes of cluster differences were represented by Cohen's d and odds ratio. In addition, multivariate logistic regression models were performed to test the associations between cluster membership and mental/behavioral health outcomes, adjusted for all sociodemographic variables. The adjusted odds ratios (AOR) and corresponding 95% confidence intervals (CI) were reported. *P*-values lower than 0.05 are considered statistically significant.

## Results

### Descriptive Characteristics

The mean age of the participants was 13.6 years and 51.9% were females. Of all the participants, 73.0% lived with both parents while 21.4% lived in a single-parent family; 15.8% of the participants' mothers and 13.4% of their fathers had obtained an educational level of college or above (Table 1). The mean values and standardized deviations for the cluster indicators, including stressful life events across six domains, self-esteem, family and friend support, maladaptive coping, and adaptive coping, are also presented in Table 1.

**Table 1:** Comparison between the clusters in sociodemographic and psychosocial variables.

**Independent variable*s***	**Total (***n*** = 3,087)**	**Cluster 1 (***n*** = 1,457)**	**Cluster 2 (***n*** = 1,106)**	**Cluster 3 (***n*** = 524)**	***P*** **-value**	**Cluster 1 vs. 2**		**Cluster 1 vs. 3**		**Cluster 2 vs. 3**	
	***N*** **(%)**	***N*** **(%)**	***N*** **(%)**	***N*** **(%)**		***P*** **-value**	**Odds Ratio**	***P*** **-value**	**Odds Ratio**	***P*** **-value**	**Odds Ratio**
**Sociodemographic variables**											
Gender					**0.012**	**0.003**		0.242		0.272	
Male	1,507 (48.1%)	736 (50.6%)	492 (44.7%)	248 (47.6%)			Ref		Ref		Ref
Female	1,629 (51.9%)	719 (49.4%)	609 (55.3%)	273 (52.4%)			1.25		1.12		0.89
Born in Hong Kong					**0.008**	**0.009**		**0.014**		0.691	
No	533 (17.0%)	216 (14.9%)	207 (18.8%)	102 (19.6%)			Ref		Ref		Ref
Yes	2,602 (83.0%)	1,236 (85.1%)	895 (81.2%)	419 (80.4%)			0.81		0.81		0.99
Living with parents					**<0.001**	**<0.001**		**<0.001**		**0.047**	
Both	2,227 (73.0%)	1,135 (78.7%)	767 (69.7%)	329 (63.8%)			Ref		Ref		Ref
Mother	504 (16.2%)	192 (13.3%)	193 (17.5%)	112 (21.7%)			1.46		1.82		1.28
Father	166 (5.3%)	58 (4.0%)	69 (6.3%)	37 (7.2%)			1.65		2.31		1.40
Neither	171 (5.3%)	58 (4.0%)	71 (6.5%)	38 (7.4%)			1.72		2.31		1.33
Mother's educational level					0.825	0.574		0.958		0.635	
Primary school or below	226 (7.3%)	87 (6.1%)	85 (7.8%)	51 (9.9%)			Ref		Ref		Ref
Middle school	1,396 (45.3%)	650 (45.7%)	490 (45.1%)	228 (44.3%)			0.87		0.74		0.83
College or undergraduate	408 (13.2%)	217 (15.3%)	128 (11.8%)	52 (10.1%)			0.73		0.58		0.78
Master or above	78 (2.5%)	41 (2.9%)	20 (1.8%)	15 (2.9%)			0.52		0.81		1.61
NA (e.g., don't know)	972 (30.9%)	426 (30.0%)	364 (33.5%)	169 (32.8%)			0.94		1.06		0.98
Father's educational level					0.110	0.903		0.054		0.052	
Primary school or below	314 (10.2%)	112 (7.8%)	124 (11.4%)	73 (14.1%)			Ref		Ref		Ref
Middle school	1,449 (46.8%)	685 (47.9%)	488 (44.8%)	243 (47.0%)			0.72		0.64		0.91
College or undergraduate	370 (11.8%)	198 (13.8%)	117 (10.7%)	49 (9.5%)			0.66		0.52		0.78
Master or above	47 (1.5%)	17 (1.2%)	19 (1.7%)	9 (1.7%)			1.53		0.93		0.65
NA (e.g., don't know)	913 (29.5%)	418 (29.2%)	341 (31.3%)	143 (27.7%)			0.72		0.44		0.67
	**Mean (SD)**	**Mean (SD)**	**Mean (SD)**	**Mean (SD)**	***P*** **-value**	***p*** **-value**	**Cohen's d**	***p*** **-value**	**Cohen's d**	***p*** **-value**	**Cohen's d**
Age	13.6 (1.3)	13.6 (1.3)	13.7 (1.3)	13.6 (1.4)	**0.007**	**0.007**	−0.08	0.562	0.00	**0.010**	0.07
**Psychological variables**											
Self-esteem	14.2 (3.0)	15.5 (2.4)	13.2 (2.8)	12.4 (3.2)	**<0.001**	**<0.001**	0.88	**<0.001**	1.10	**<0.001**	0.27
Family support	11.1 (5.1)	13.3 (4.5)	9.4 (4.8)	8.5 (5.0)	**<0.001**	**<0.001**	0.84	**<0.001**	1.01	**<0.001**	0.18
Friend support	10.6 (5.0)	12.1 (4.6)	9.3 (4.7)	9.1 (5.2)	**<0.001**	**<0.001**	0.60	**<0.001**	0.60	0.448	0.04
Adaptive coping	16.3 (3.2)	16.0 (3.3)	16.3 (2.9)	17.2 (3.4)	**<0.001**	**0.022**	−0.10	**<0.001**	−0.36	**<0.001**	−0.29
Maladaptive coping	12.9 (2.6)	12.0 (2.5)	13.3 (2.4)	14.4 (2.7)	**<0.001**	**<0.001**	−0.53	**<0.001**	−0.92	**<0.001**	−0.43
SLE-relationship	4.7 (4.3)	1.7 (1.8)	5.6 (3.0)	11.3 (3.6)	**<0.001**	**<0.001**	−1.58	**<0.001**	−3.37	**<0.001**	−1.72
SLE-academic pressure	4.4 (4.0)	1.8 (1.8)	5.1 (2.8)	10.4 (3.7)	**<0.001**	**<0.001**	−1.40	**<0.001**	−2.96	**<0.001**	−1.62
SLE-punishment	3.4 (4.8)	0.6 (1.1)	3.3 (2.7)	11.5 (5.3)	**<0.001**	**<0.001**	−1.31	**<0.001**	−2.85	**<0.001**	−1.95
SLE-bereavement	1.5 (2.6)	0.3 (0.9)	1.6 (2.1)	4.8 (3.6)	**<0.001**	**<0.001**	−0.81	**<0.001**	−1.72	**<0.001**	−1.09
SLE-health and adaption	1.5 (2.0)	0.5 (0.8)	1.6 (1.5)	4.2 (2.5)	**<0.001**	**<0.001**	−0.92	**<0.001**	−1.99	**<0.001**	−1.26
SLE-others	2.2 (2.7)	0.6 (1.0)	2.4 (1.8)	6.5 (2.9)	**<0.001**	**<0.001**	−1.24	**<0.001**	−2.72	**<0.001**	−1.70

*Post-hoc LSD significant comparison (0.05). SLE, stressful life events. P-values < 0.05 are presented in bold*.

### Clustering Outcomes

**Figure 1:**
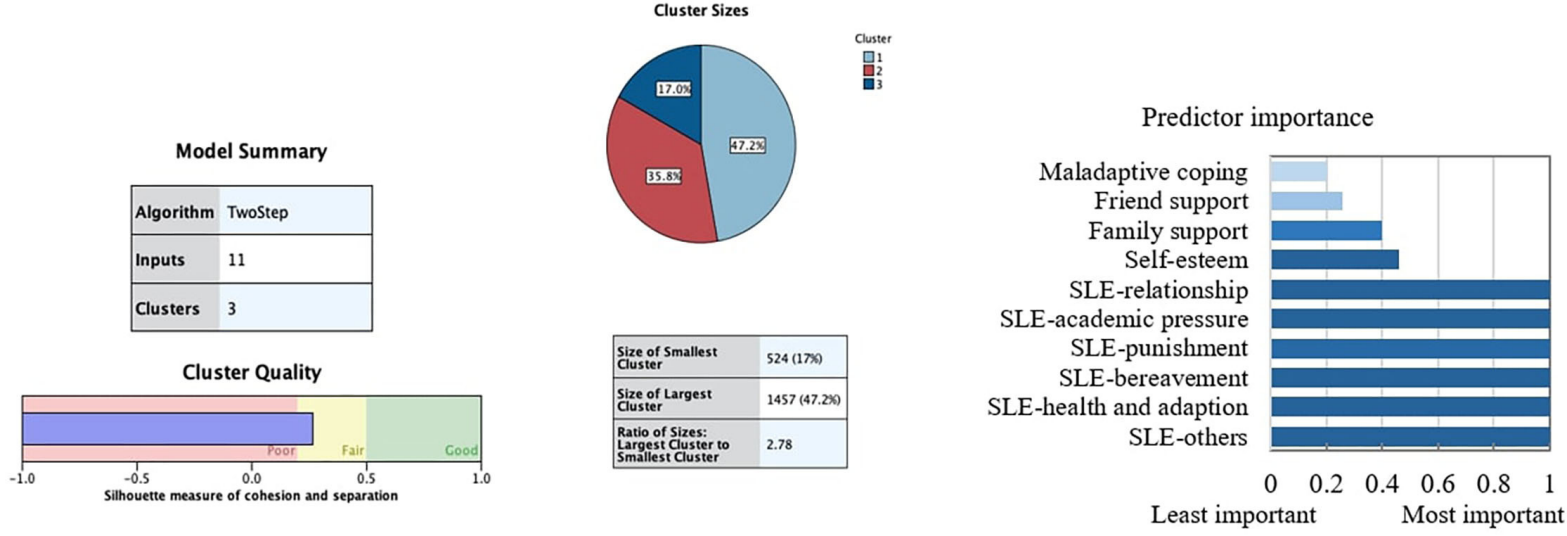
Summary of clustering results. SLE, stressful life events.

The solution selected as the optimal in the two-step cluster procedure was for three latent empirical groups. As shown in Supplementary Table 1, this solution gave the highest value for the ratio of distance measures and lower value of BIC. The 3-cluster solution achieved the highest ratio distance measure (2.78) and a cohesion/ separation index into the fair range (silhouette = 0.3). Figure 1 displays the ordered bar chart with the relative relevance for each indicator used in the clustering. These weights ranged from 0 to 1, and greater values indicate a lower likelihood that changes between clusters are attributable to chance. The measures with the greatest discriminative relevance were the six subscales of stressful life events, followed by self-esteem, family support, maladaptive coping, and friend support.

### Comparison Between Clusters

**Figure 2:**
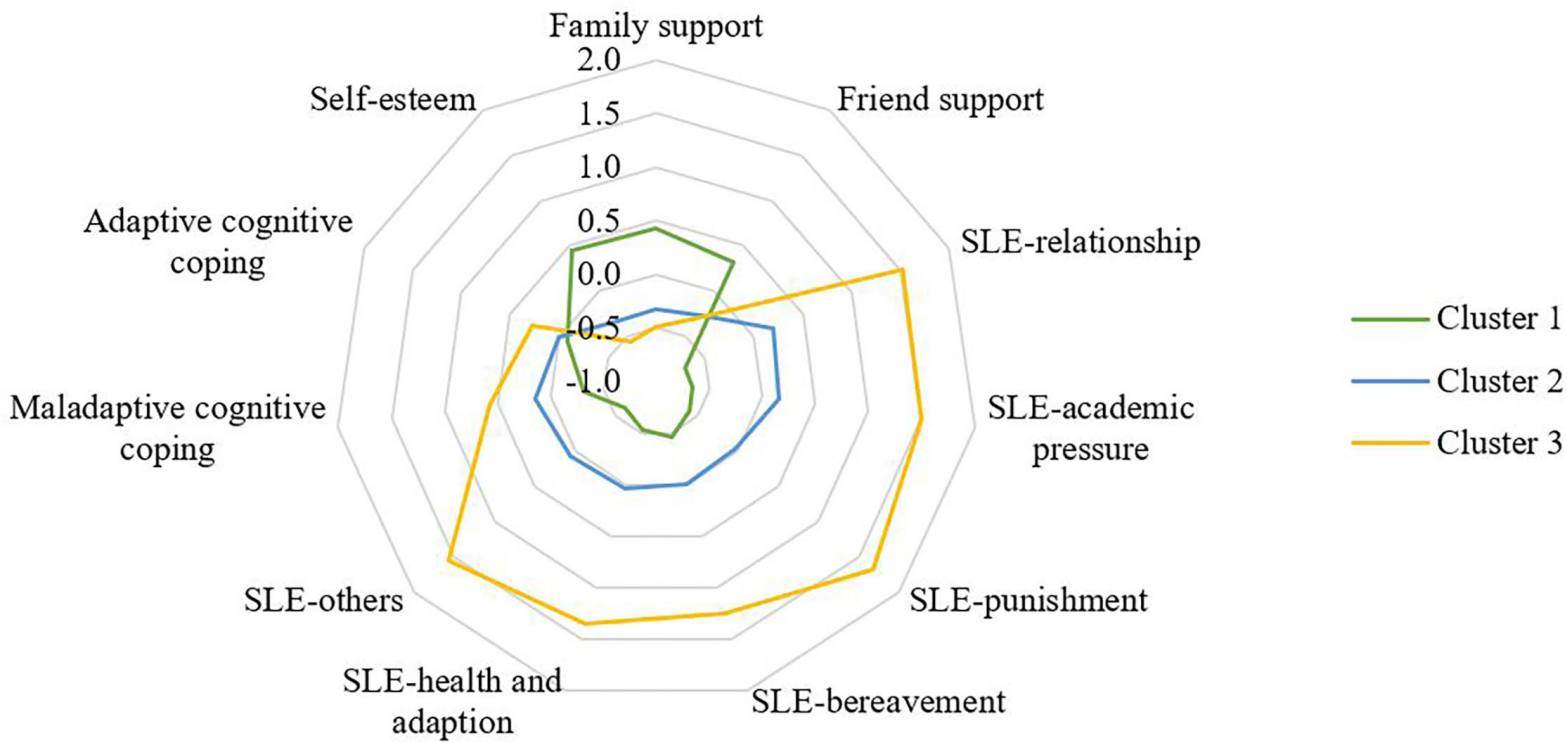
Radar-chart displaying the differences between the three clusters. SLE, stressful life events. Standardized scale scores were presented.

**Figure 3:**
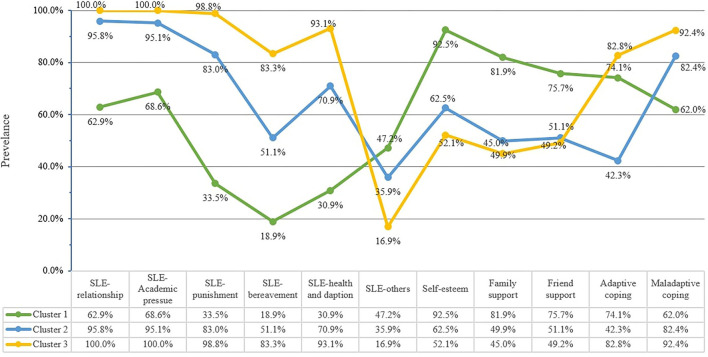
Line-chart displaying the levels of participants' risk and protective profiles. SLE: stressful life events. The percentage of SLE variables represented the proportion of individuals who had at least one type of stressful life events in the specified domain. The percentage of self-esteem, family support, friend support, adaptive coping and maladaptive coping variables represented the proportion of individuals who scored above the median of the specified scale.

Descriptive data for sociodemographic characteristics, stressful life events, self-esteem, family support, friend support, maladaptive coping, and adaptive coping are reported by cluster in Table 1 and showed graphically in Figures 2, 3. The largest cluster (47.2%) was characterized by low levels of stressful life events and maladaptive and adaptive coping as well as high levels of self-esteem, family support, and friend support, compared to the remaining two clusters. Cluster 2 comprised 35.8% of the sample, characterized by moderate levels of stressful life events, coping strategies, self-esteem, and perceived support. The final cluster 3 was marked by high values on all six domains of stressful life events, high levels of maladaptive and adaptive coping, and low levels of self-esteem, family support, and friend support. The three clusters differed significantly in the levels of these indicators and the majority showed a large effect size of between-group differences (Cohen's *d* > 0.8; Table 1). Based on the set of results in this study, cluster 1 was labeled as “low-risk profile (Low Risk/Low Protective)”, cluster 2 as “moderate-risk profile (Moderate Risk/Moderate Protective)”, and cluster 3 as “severe-risk profile (High Risk/Low Protective)”.

With respect to sociodemographic variables, the three clusters significantly differed in gender, age, whether or not born in Hong Kong, and living arrangement. Participants who were born in Hong Kong and lived with both parents were more likely to be classified into the low profile group (cluster 1). Males and younger adolescents were over-represented in cluster 1 relative to cluster 2 (Table 1).

### Associations Between Cluster Membership and Mental, Behavioral, and Internet Use Problems

**Figure 4:**
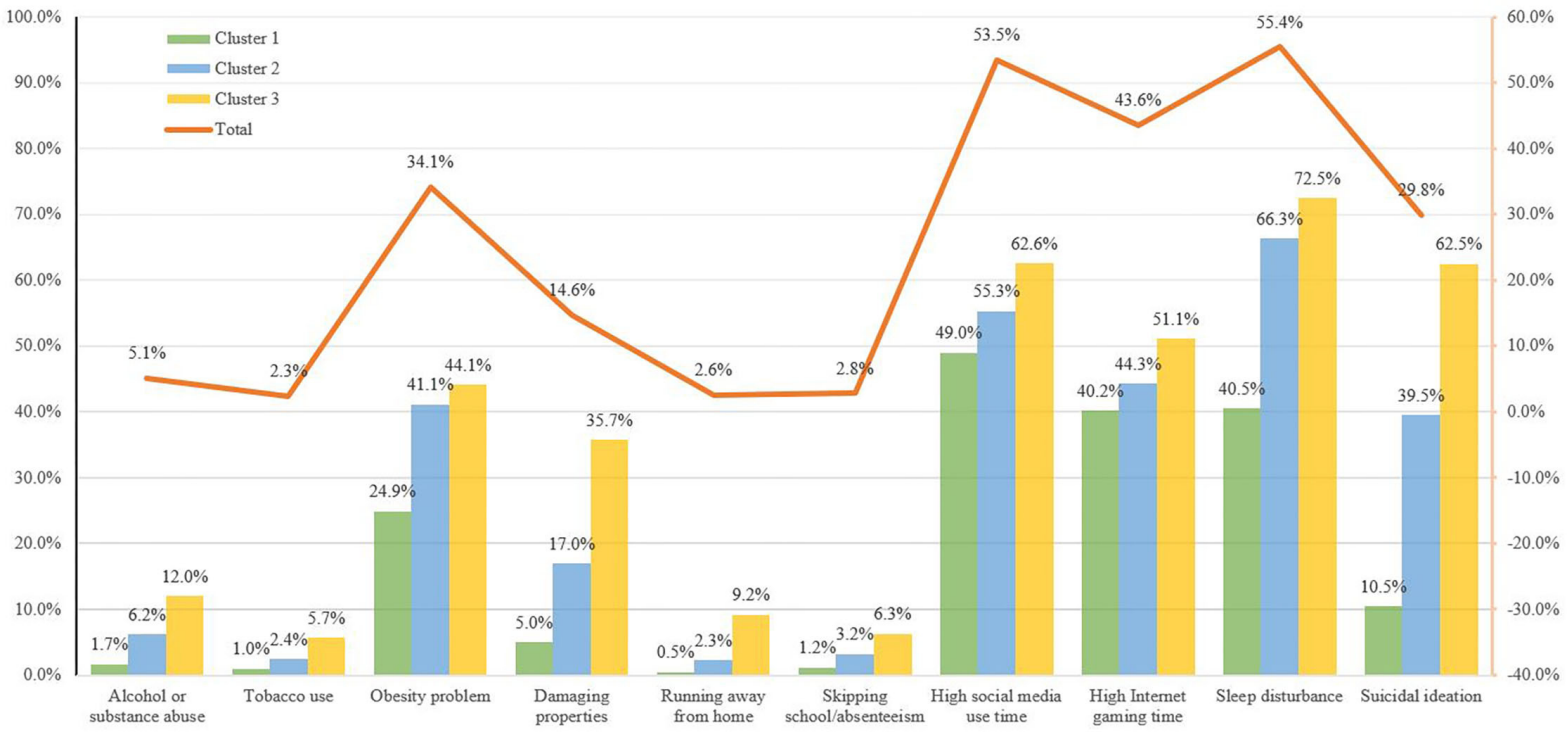
Frequency of behavioral, Internet use, and mental health problems by clusters.

Figure 4 illustrates the prevalence of mental, behavioral, and Internet use problems in the total sample and by clusters. 5.1, 2.3, 2.6, and 2.8% of the participants reported that they had alcohol or substance abuse, tobacco use, running away from home, and skipping school/absenteeism in the previous 6 months, respectively. While about half of the participants reported that they had excessive social media and Internet gaming use and 34.1% had obesity problems. Concerning mental health, 29.8% of them reported suicidal ideation in the past 2 weeks and half of them had sleep disturbance in the past 6 months (55.4%). The detailed frequency distribution of mental, behavioral, and Internet use problems is presented in Supplementary Table 2.

Adjusted for all sociodemographic variables, the multivariate logistic regression models indicated that compared to the low profile cluster, individuals in the moderate and severe profile groups consistently reported significantly higher prevalence of behavioral (range for AOR: 1.22–11.22; all *p* < 0.05) and mental health problems (range for AOR: 2.08–15.06; all *p* < 0.001) (Table 2). We also conducted multinominal logistic regression analysis to assess the associations between cluster membership and behavioral/mental health outcomes using the original response categories of dependent variables, which showed similar significant results (Supplementary Table 3).

**Table 2:** Associations between cluster membership and behavioral, Internet use, and mental health problems.

**Dependent outcomes**		**AOR (95% CI)**	***P*** **-value**
Alcohol or substance abuse	Cluster 1 (ref)	1.00	
	Cluster 2	3.26 (2.03, 5.25)***;	<0.001
	Cluster 3	6.92 (4.23, 11.32)***;	<0.001
Tobacco use	Cluster 1 (ref)	1.00	
	Cluster 2	2.05 (1.07, 3.93)*	0.032
	Cluster 3	5.14 (2.69, 9.84)***;	<0.001
Obesity	Cluster 1 (ref)	1.00	
	Cluster 2	2.08 (1.74, 2.49)***;	<0.001
	Cluster 3	2.42 (1.93, 3.02)***;	<0.001
Damaging properties	Cluster 1 (ref)	1.00	
	Cluster 2	4.17 (3.08, 5.63)***;	<0.001
	Cluster 3	11.22 (8.17, 15.39)***;	<0.001
Running away from home	Cluster 1 (ref)	1.00	
	Cluster 2	4.47 (1.91, 10.46)***;	<0.001
	Cluster 3	17.95 (7.95, 40.51)***;	<0.001
Skipping school/absenteeism	Cluster 1 (ref)	1.00	
	Cluster 2	2.82 (1.52, 5.24)**	0.001
	Cluster 3	4.92 (2.57, 9.40)***;	<0.001
Excessive social media use	Cluster 1 (ref)	1.00	
	Cluster 2	1.19 (1.01, 1.41)*	0.041
	Cluster 3	1.75 (1.40, 2.19)***;	<0.001
Excessive Internet gaming use	Cluster 1 (ref)	1.00	
	Cluster 2	1.22 (1.02, 1.45)*	0.026
	Cluster 3	1.65 (1.32, 2.06)***;	<0.001
Suicidal ideation	Cluster 1 (ref)	1.00	
	Cluster 2	5.80 (4.66, 7.23)***;	<0.001
	Cluster 3	15.06 (11.59, 19.56)***;	<0.001
Sleep disturbance	Cluster 1 (ref)	1.00	
	Cluster 2	2.66 (2.24, 3.16)***;	<0.001
	Cluster 3	3.82 (3.03, 4.82)***;	<0.001

* *p < 0.05*,

** *p < 0.01*,

*** *p < 0.001*.

## Discussion

### Principal Results

The present study investigated the prevalence of various mental, behavioral, and Internet use problems in a large-scale adolescent population in Hong Kong, China, during the COVID-19 pandemic. We further explored whether adolescents could be differentiated based on stress and coping profiles, and assessed whether their levels of health-related behaviors and mental health problems varied as a function of these profiles. The results provided empirical evidence that adolescents' behavioral and mental health may be shaped by the combined profiles of their stressful experiences, coping resources, and adopted coping strategies. These findings highlight the potential for multimodal stress and coping interventions that may help adolescents to prevent and reduce their various psychological and behavioral risks.

We found that about 30% of the adolescents expressed suicidal ideation and half of them had sleep disturbance, which was higher than the prevalence of suicidal ideation reported in a pre-COVID-19 study among a representative sample of secondary school students in Hong Kong (13.7%) (38). Other studies also reported significant increases in mental health problems among adolescents due to the COVID-19 pandemic (39, 40). The prevalence of problematic behaviors in the present study is comparable to or lower than the findings of a pre-COVID-19 study among 7,975 Hong Kong secondary school students, including smoking (5.1 vs. 7.8%), substance use (2.3 vs. 2.5%), running away from home (2.6 vs. 6.7%), truancy (2.8 vs. 6.2%), and damaging properties (11.4 vs. 14.5%) (41). This pattern corroborates a prior national survey in Iceland, showing that COVID-19 has significantly increased the mental distress of adolescents while the decrease in risky behaviors (e.g., substance use) was observed (42). It is plausible that the COVID-19 preventive measures (e.g., social isolation) have reduced adolescents' access to substances or risky behaviors. This is also consistent with social developmental theories that adolescents' behaviors are socially influenced, and preventive measures might have reduced negative peer influence on and rewards of exploring problematic behaviors (43). In addition, parental supervision over children's behaviors may have enhanced due to school closure and increased time spent at home. Notably, of the various problematic behaviors, adolescents reported the highest prevalence in excessive Internet gaming and social media use, and obesity (all >30%). Similarly, a previous study also suggested that adolescents' Internet gaming increased during the pandemic and school closures (44). These findings underline the need for interventions of adolescents' sedentary lifestyles especially Internet use during the pandemic to prevent the potential long-term adverse health impact. Continued parental surveillance of Internet use behaviors at different stages of the pandemic and after schools are resumed is greatly warranted.

Based on the multivariate stressful experiences, coping resources, and coping responses, participants were categorized into three groups: High Risk/Low Protective, Moderate Risk/Moderate Protective, and Low Risk/High Protective. The two-step cluster analysis indicated that a three-cluster solution was the best model for a fair cluster quality, evidenced by the lower value of BIC and the maximum ratio of distance measures (45). Future studies are warranted to confirm whether this clustering pattern exists among adolescents in different regions. We found that adolescents who were not born in Hong Kong and in single-family were more likely to have moderate/severe profiles, supporting the migration morbidity hypothesis that immigration leads to psychosocial problems in migrant populations (46). Such at-risk subpopulations warrant further investigation. While low-risk group reported the highest levels of self-esteem, family support, and friend support, which might be protective factors of adolescents' mental and behavioral health (9, 17). Furthermore, clustering results supported that adolescents with high levels of stressful life events had relatively low self-esteem, social support as well as more maladaptive and adaptive coping. This pattern corroborates previous studies that stressful experiences may lead to feelings of powerlessness, helplessness, and loss of self-control, which thus result in a predisposition toward more maladaptive coping (47). The availability of coping resources may also inhibit stress proliferation indirectly through its effect on the choice of coping strategy (e.g., less maladaptive coping). Unexpectedly, maladaptive coping showed a similar pattern with adaptive coping across clusters. This may suggest that maladaptive and adaptive coping are relatively two distinct constructs instead of the opposite ends of a single dimension (48). It is plausible that people use more than one strategy, including both maladaptive and adaptive styles, to manage a stressful situation. In addition, adaptive coping showed a relatively weaker discriminative relevance in identifying at-risk groups compared to maladaptive coping, corroborating previous studies that maladaptive coping has stronger associations with health outcomes than adaptive coping (48). Relevant stakeholders including educators, gatekeepers, and parents should be made aware of adolescents' coping characteristics and skill training about the reduction of maladaptive coping and promotion of adaptive coping may be considered as an important agenda in health policy and health education in advancing adolescents' mental and behavioral health.

Beyond identifying stress-coping profiles using cluster analysis, the present study found consistent and robust associations between cluster membership and a wide range of mental and behavioral health problems. Compared to the low-risk cluster, individuals with high and moderate profiles reported a significantly higher prevalence of problematic behaviors and mental health issues. Notably, the dose-response relationship was observed that the severe cluster had larger odds ratios than the moderate cluster across all health outcomes when using the low-risk cluster as the reference group. These findings provide empirical support for the stress-coping theory that stressful experiences and coping strategies play significant roles in determining individuals' mental and behavioral health (11, 13, 14, 17). This study further demonstrated a deeper understanding that adolescents' behaviors and psychological health may be shaped by their combined profiles of stressful experiences, available coping resources, and coping styles. Relevant health promotions may incorporate and target different perspectives of stress and coping to achieve greater intervention efficacy.

### Limitations

The study has limitations. First, the cross-sectional design cannot determine causal inferences. Adolescents with mental distress might further isolate themselves, resulting in reduced social support and increased maladaptive coping. Future prospective studies are warranted. Second, self-reported measures might be subject to recall bias and social desirability bias (e.g., disclosing substance use may be sensitive for students). In order to increase response rate and guarantee response quality, short measures of coping and self-esteem were used. Maladaptive and adaptive coping subscales showed low internal reliability. Future studies should be replicated with the full CERQ scale and further test the appropriateness of two-factor structure (i.e., adaptive and maladaptive dimensions) using confirmatory factor analysis. Third, as data for this study were gathered from young adolescents from participating secondary schools in Hong Kong, there might be selection bias. Findings cannot be generalized to out-of-school adolescents, e.g., institutionalized adolescents, home-schooled adolescents, or adolescents who cannot attend school in-person due to/during the pandemic. The generalization of the findings should be cautious and future study may consider stratified random sampling based on the number of schools in each district to improve the representativeness of the sampling. However, the sex and age distribution in the current sample was comparable to the latest census data of secondary grade 1–4 students in Hong Kong (49). Fourth, we assessed obesity problem solely using previous behavioral scale item. The measurement of physical activity, sedentary behavior, and body mass index may be considered in future studies to have a more comprehensive investigation of adolescents' physical health and health behaviors. Fifth, the silhouette coefficient of clustering result indicated a fair model fit. Robust clustering methodology with better fit and taking into account outliers should be explored. Lastly, future studies should include other important mental health problems (e.g., depression and anxiety) and problematic behaviors (e.g., stealing and bullying), and explore the interplay between mental and behavioral risks among adolescents.

## Conclusions and Public Mental Health Implications

This study is a timely investigation of mental and behavioral health problems among Chinese adolescents in Hong Kong during the COVID-19 pandemic, and efforts should be made to mitigate adolescents' mental and behavioral health problems. The findings offer important public health and clinical implications in the assessment and potential management of at-risk adolescents. First, high levels of stressful life events were reported by adolescents, especially in the domain of relationships, academics, or being punished. Pre-COVID studies also suggested that adolescents' stressful life events mainly came from academic domain and interpersonal relationships with family, teachers, and friends (8, 50). School social workers and counselors should be aware of these common stressors of adolescents. Second, students at risk of suffering from mental distress and engaging in unhealthy behaviors could be systematically screened primarily based on their profiles of negative life events as these indicators provided the greatest discriminative power to identify different clusters. However, coping resources and strategies may provide additional precision to identify groups that might best benefit from interventions in a cost-effective manner (12). Health promotions to reduce adolescents' mental and behavioral risks should thus incorporate and target both stress and coping to achieve greater intervention efficacy, such as alleviating stressful perceptions, increasing coping resources (e.g., enhancing social support and self-efficacy), and reducing maladaptive coping. Therefore, interventions based on transdiagnostic approaches, such as cognitive-behavioral therapy and problem-solving therapy, and the formation of supportive networks for adolescents, may be particularly beneficial and efficient to prevent and reduce multiple mental and behavioral risks in a cost-effective manner (37).

## Data Availability Statement

The raw data supporting the conclusions of this article will be made available by the authors, without undue reservation.

## Ethics Statement

Ethics approval was obtained from the Survey and Behavioral Ethics Committee of the corresponding author's affiliated institution (Ref No. SBRE-18-433). Written informed consent to participate in this study was provided by the participants' legal guardian/next of kin.

## Author Contributions

RS: conceptualization, methodology, and writing—original draft. KW, JL, and YZ: data curation. RS and JL: formal analysis. XY: funding acquisition and writing—review and editing. KW: investigation. All authors commented on previous versions of the manuscript and approved the final manuscript.

## Funding

This study was funded by the Health and Medical Research Fund (#16171001) and General Research Fund (#6905082). The funder had no involvement in the study design and collection, analysis, and interpretation of the results.

## Conflict of Interest

The authors declare that the research was conducted in the absence of any commercial or financial relationships that could be construed as a potential conflict of interest.

## Publisher's Note

All claims expressed in this article are solely those of the authors and do not necessarily represent those of their affiliated organizations, or those of the publisher, the editors and the reviewers. Any product that may be evaluated in this article, or claim that may be made by its manufacturer, is not guaranteed or endorsed by the publisher.

## References

[1] Kieling C Baker-Henningham H Belfer M Conti G Ertem I Omigbodun O. Child and adolescent mental health worldwide: evidence for action. Lancet. (2011) 378:1515–25. 10.1016/S0140-6736(11)60827-1 22008427

[2] Ghandour RM Sherman LJ Vladutiu CJ Ali MM Lynch SE Bitsko RH. Prevalence and treatment of depression, anxiety, and conduct problems in US children. J Pediatr. (2019) 206:256–67. e3. 10.1016/j.jpeds.2018.09.021 30322701 PMC6673640

[3] Yang X Hu H Zhao C Xu H Tu X Zhang G. longitudinal study of changes in smart phone addiction and depressive symptoms and potential risk factors among Chinese college students. BMC Psychiatry. (2021) 21:1–9. 10.1186/s12888-021-03265-4 33990181 PMC8120756

[4] Panda PK Gupta J Chowdhury SR Kumar R Meena AK Madaan P. Psychological and behavioral impact of lockdown and quarantine measures for COVID-19 pandemic on children, adolescents and caregivers: a systematic review and meta-analysis. J Trop Pediatr. (2021) 67:fmaa122. 10.1093/tropej/fmaa122 33367907 PMC7798512

[5] Dong H Yang F Lu X Hao W. Internet addiction and related psychological factors among children and adolescents in China during the coronavirus disease 2019 (COVID-19) epidemic. Front Psychiatry. (2020) 11:751. 10.3389/fpsyt.2020.00751 32982806 PMC7492537

[6] Teng Z Pontes HM Nie Q Griffiths MD Guo C. Depression and anxiety symptoms associated with internet gaming disorder before and during the COVID-19 pandemic: a longitudinal study. J Behav Addict. (2021) 10:169–80. 10.1556/2006.2021.00016 33704085 PMC8969853

[7] She R Yang X Lau MMC Lau JTF. Psychometric properties and normative data of the 10-item Connor–Davidson Resilience Scale among Chinese adolescent students in Hong Kong. Child Psychiatry Hum Dev. (2020) 51:925–33. 10.1007/s10578-020-00970-1 32086664

[8] Liu X Kurita H Uchiyama M Okawa M Liu L Ma D. Life events, locus of control, and behavioral problems among Chinese adolescents. J Clin Psychol. (2000) 56:1565–77. 10.1002/1097-4679(200012)56:12<1565::AID-7>3.0.CO;2-U 11132571

[9] Tandon SD Dariotis JK Tucker MG Sonenstein FL. Coping, stress, and social support associations with internalizing and externalizing behavior among urban adolescents and young adults: revelations from a cluster analysis. J Adolesc Health. (2013) 52:627–33. 10.1016/j.jadohealth.2012.10.001 23298992

[10] Lazarus RS Folkman S. Stress, Appraisal, and Coping. New York, NY: Springer Publishing Company (1984).

[11] Huh HJ Kim KH Lee H-K Chae J-H. The relationship between childhood trauma and the severity of adulthood depression and anxiety symptoms in a clinical sample: the mediating role of cognitive emotion regulation strategies. J Affect Disord. (2017) 213:44–50. 10.1016/j.jad.2017.02.009 28189964

[12] Doron J Trouillet R Maneveau A Neveu D Ninot G. Coping profiles, perceived stress and health-related behaviors: a cluster analysis approach. Health Promot Int. (2015) 30:88–100. 10.1093/heapro/dau090 25324530

[13] Lei H Cheong CM Li S Minghui L. The relationship between coping style and Internet addiction among mainland Chinese students: a meta-analysis. Psychiatry Res. (2018) 270:831–41. 10.1016/j.psychres.2018.10.079 30551332

[14] Horwitz AG Hill RM King CA. Specific coping behaviors in relation to adolescent depression and suicidal ideation. J Adolesc. (2011) 34:1077–85. 10.1016/j.adolescence.2010.10.004 21074841 PMC3319342

[15] She R Wong K Lin J Leung K Zhang Y Yang X. How COVID-19 stress related to schooling and online learning affects adolescent depression and Internet gaming disorder: testing conservation of resources theory with sex difference. J Behav Addict. (2021). 10.1556/2006.2021.00069. [Epub ahead of print]. 34665762 PMC8987435

[16] Terry DJ. Coping resources and situational appraisals as predictors of coping behavior. Pers Individ Dif. (1991) 12:1031–47. 10.1016/0191-8869(91)90033-8

[17] Siyez DM. Adolescent self-esteem, problem behaviors, and perceived social support in Turkey. Soc Behav Person Int J. (2008) 36:973–84. 10.2224/sbp.2008.36.7.97317992826

[18] Ellis A. Individual and Interactive Effects of Childhood Problem Behaviors and Maternal Discipline on Adolescent Problem Behavior and Alcohol Use. Ypsilanti, MI: Eastern Michigan University (2012).

[19] Jacobs JE Vernon MK Eccles JS. Relations between social self-perceptions, time use, and prosocial or problem behaviors during adolescence. J Adolesc Res. (2004) 19:45–62. 10.1177/0743558403258225

[20] Stavridou A Kapsali E Panagouli E Thirios A Polychronis K Bacopoulou F. Obesity in children and adolescents during COVID-19 pandemic. Children. (2021) 8:135. 10.3390/children8020135 33673078 PMC7918914

[21] Achenbach TM Edelbrock C. Child behavior checklist. Burlington. (1991) 7:371–92.

[22] Gentile DA Bailey K Bavelier D Brockmyer JF Cash H Coyne SM. Internet gaming disorder in children and adolescents. Pediatrics. (2017) 140(Suppl. 2):S81–5. 10.1542/peds.2016-1758H 29093038

[23] Kroenke K Spitzer RL. Williams JBW. The PHQ-9. J Gen Intern Med. (2001) 16:606–13. 10.1046/j.1525-1497.2001.016009606.x 11556941 PMC1495268

[24] Rossom RC Coleman KJ Ahmedani BK Beck A Johnson E Oliver M. Suicidal ideation reported on the PHQ9 and risk of suicidal behavior across age groups. J Affect Disord. (2017) 215:77–84. 10.1016/j.jad.2017.03.037 28319695 PMC5412508

[25] Yang X Jiang X Mo P Cai Y Ma L Lau J. Prevalence and interpersonal correlates of internet gaming disorders among Chinese adolescents. Int J Environ Res Public Health. (2020) 17:579. 10.3390/ijerph17020579 31963197 PMC7013587

[26] Monteiro RP Coelho GLdH Hanel PHP de Medeiros ED da Silva PDG. The efficient assessment of self-esteem: proposing the brief rosenberg self-esteem scale. Appl Res Qual Life. (2021). 10.1007/s11482-021-09936-4. [Epub ahead of print].

[27] Garnefski N Kraaij V. Cognitive emotion regulation questionnaire–development of a short 18-item version (CERQ-short). Pers Individ Dif. (2006) 41:1045–53. 10.1016/j.paid.2006.04.010

[28] Zhu X Auerbach RP Yao S Abela JR Xiao J Tong X. Psychometric properties of the cognitive emotion regulation questionnaire: Chinese version. Cogn Emot. (2008) 22:288–307. 10.1080/0269993070136903526925586

[29] Ding F Wang X Cheng C He J Zhao H Wu D. Psychometric properties and measurement invariance of the cognitive emotion regulation questionnaire in chinese adolescents with and without major depressive disorder: a horizontal and longitudinal perspective. Front Psychiatry. (2021) 12:736887. 10.3389/fpsyt.2021.736887 34744827 PMC8569313

[30] Orgilés M Morales A Fernández-Martínez I Melero S Espada JP. Validation of the short version of the cognitive emotion regulation questionnaire for Spanish children. J Child Health Care. (2019) 23:87–101. 10.1177/1367493518777306 29788778

[31] Santos AC Simões C Daniel JR Arriaga P. Portuguese validation of the cognitive emotion regulation questionnaire short version in youth: validity, reliability and invariance across gender and age. Eur J Dev Psychol. (2021). 10.1080/17405629.2021.2011201. [Epub ahead of print].

[32] She R Luo S Lau MM Lau JTF. The mechanisms between illness representations of COVID-19 and behavioral intention to visit hospitals for scheduled medical consultations in a Chinese general population. J Health Psychol. (2021). 10.1177/13591053211008217 33878946

[33] Yusoff MSB. Stability of DREEM in a sample of medical students: a prospective study. Educ Res Int. (2012) 2012:509638. 10.1155/2012/509638

[34] Benassi M Garofalo S Ambrosini F Sant'Angelo RP Raggini R De Paoli G. Using two-step cluster analysis and latent class cluster analysis to classify the cognitive heterogeneity of cross-diagnostic psychiatric inpatients. Front Psychol. (2020) 11:85. 10.3389/fpsyg.2020.01085 32587546 PMC7299079

[35] Gelbard R Goldman O Spiegler I. Investigating diversity of clustering methods: an empirical comparison. Data Knowl Eng. (2007) 63:155–66. 10.1016/j.datak.2007.01.002

[36] Marques DR Gomes AA Clemente V Drake CL Roth T Morin CM. Typologies of individuals vulnerable to insomnia: a two-step cluster analysis. Sleep Biol Rhythms. (2021) 19:33–44. 10.1007/s41105-020-00285-7

[37] Munguía L Jiménez-Murcia S Granero R Baenas I Agüera Z Sánchez I. Emotional regulation in eating disorders and gambling disorder: a transdiagnostic approach. J Behav Addict. (2021) 41:1045–53. 10.1556/2006.2021.00017 33784249 PMC8997225

[38] Shek DTL Yu L. Self-harm and suicidal behaviors in hong kong adolescents: prevalence and psychosocial correlates. Sci World J. (2012) 2012:932540. 10.1100/2012/932540 22566783 PMC3322490

[39] Magson NR Freeman JYA Rapee RM Richardson CE Oar EL Fardouly J. Risk and protective factors for prospective changes in adolescent mental health during the COVID-19 pandemic. J Youth Adolesc. (2021) 50:44–57. 10.1007/s10964-020-01332-9 33108542 PMC7590912

[40] Cost KT Crosbie J Anagnostou E Birken CS Charach A Monga S. Mostly worse, occasionally better: impact of COVID-19 pandemic on the mental health of Canadian children and adolescents. Eur Child Adolesc Psychiatry. (2021). 10.1007/s00787-021-01744-3. [Epub ahead of print]. 33638005 PMC7909377

[41] Sun RC Shek DT. Life satisfaction, positive youth development, and problem behaviour among Chinese adolescents in Hong Kong. Soc Indic Res. (2010) 95:455–74. 10.1007/s11205-009-9531-9 20062815 PMC2801834

[42] Thorisdottir IE Asgeirsdottir BB Kristjansson AL Valdimarsdottir HB Jonsdottir Tolgyes EM Sigfusson J. Depressive symptoms, mental wellbeing, and substance use among adolescents before and during the COVID-19 pandemic in Iceland: a longitudinal, population-based study. Lancet Psychiatry. (2021) 8:663–72. 10.1016/S2215-0366(21)00156-5 34090582

[43] Allen JP Loeb EL Narr RK Costello MA. Different factors predict adolescent substance use versus adult substance abuse: lessons from a social-developmental approach. Dev Psychopathol. (2020) 33:792–802. 10.1017/S095457942000005X 32638695 PMC7755752

[44] Zhu S Zhuang Y Lee P Li JC-M Wong PW. Leisure and problem gaming behaviors among children and adolescents during school closures caused by COVID-19 in Hong Kong: quantitative cross-sectional survey study. JMIR Serious Games. (2021) 9:e26808. 10.2196/26808 33960954 PMC8108935

[45] Sarstedt M Mooi E. A concise guide to market research. Process Data. (2014) 12:298–9. 10.1007/978-3-642-53965-7

[46] Jiang H Shek DT Law MY. Differences between Chinese adolescent immigrants and adolescent non-immigrants in Hong Kong: perceived psychosocial attributes, school environment and characteristics of Hong Kong adolescents. Int J Environ Res Public Health. (2021) 18:3739. 10.3390/ijerph18073739 33918464 PMC8038285

[47] Bandermann KM Szymanski DM. Exploring coping mediators between heterosexist oppression and posttraumatic stress symptoms among lesbian, gay, and bisexual persons. Psychol Sex Orient Gender Divers. (2014) 1:213. 10.1037/sgd0000044

[48] Moritz S Jahns AK Schröder J Berger T Lincoln TM Klein JP. More adaptive versus less maladaptive coping: what is more predictive of symptom severity? Development of a new scale to investigate coping profiles across different psychopathological syndromes. J Affect Disord. (2016) 191:300–7. 10.1016/j.jad.2015.11.027 26702520

[49] Education Bureau Government of the Hong Kong Special Administrative Region. Student Enrolment Statistics 2019/20. Hong Kong (2020).

[50] Chan DW. Stressful life events, cognitive appraisals, and psychological symptoms among Chinese adolescents in Hong Kong. J Youth Adolesc. (1998) 27:457–72. 10.1023/A:1022800118777

