# Supplementary material for: Profiles of Stress and Coping Associated With Mental, Behavioral, and Internet Use Problems Among Adolescents During the COVID-19 Pandemic: A Stratified Random Sampling and Cluster Analysis
Source: Front Public Health. 2022 Mar 29;10:826911. doi: 10.3389/fpubh.2022.826911 (PMC9002121; doi:10.3389/fpubh.2022.826911)
Supplement: Supplementary file 1 [file Table_1.docx]

Supplementary Table 1 Auto-Clustering comparison

| Number of Clusters | Schwarz's Bayesian Criterion (BIC) | BIC Change^a^ | Ratio of BIC Changes^b^ | Ratio of Distance Measures^c^ |
| --- | --- | --- | --- | --- |
| 1 | 23708.467 |  |  |  |
| 2 | 18891.977 | -4816.491 | 1.000 | 2.570 |
| 3 | 17140.769 | -1751.208 | .364 | 2.784 |
| 4 | 16630.499 | -510.270 | .106 | 1.027 |
| 5 | 16138.141 | -492.358 | .102 | 1.100 |
| 6 | 15706.527 | -431.614 | .090 | 1.457 |
| 7 | 15465.642 | -240.885 | .050 | 1.047 |
| 8 | 15243.445 | -222.196 | .046 | 1.432 |
| 9 | 15141.593 | -101.852 | .021 | 1.079 |
| 10 | 15060.071 | -81.522 | .017 | 1.112 |
| 11 | 15004.607 | -55.463 | .012 | 1.050 |
| 12 | 14960.126 | -44.482 | .009 | 1.053 |
| 13 | 14926.787 | -33.339 | .007 | 1.192 |
| 14 | 14927.225 | .438 | .000 | 1.011 |
| 15 | 14929.567 | 2.342 | .000 | 1.085 |

1. The changes are from the previous number of clusters in the table.
2. The ratios of changes are relative to the change for the two-cluster solution.
3. The ratios of distance measures are based on the current number of clusters against the previous number of clusters.

Supplementary Table 2 Frequency distribution of behavioral, Internet use, and mental health problems

|  | **Cluster 1**  N (%) | **Cluster 2**  N (%) | **Cluster 3**  N (%) | **Total**  N (%) |
| --- | --- | --- | --- | --- |
| **Alcohol or substance abuse** |  |  |  |  |
| Never | 1430 (98.3%) | 1036 (93.8%) | 460 (88.0%) | 2926 (94.9%) |
| Sometimes | 24 (1.6%) | 63 (5.7%) | 54 (10.3%) | 141 (4.6%) |
| Often | 1 (0.1%) | 6 (0.5%) | 9 (1.7%) | 16 (0.5%) |
| **Tobacco use** |  |  |  |  |
| Never | 1442 (99.0%) | 1078 (97.6%) | 493 (94.3%) | 3013 (97.7%) |
| Sometimes | 13 (0.9%) | 22 (2.0%) | 22 (4.2%) | 57 (1.8%) |
| Often | 2 (0.1%) | 5 (0.5%) | 8 (1.5%) | 15 (0.5%) |
| **Sleep disturbance** |  |  |  |  |
| Never | 867 (59.5%) | 372 (33.7%) | 144 (27.5%) | 1383 (44.8%) |
| Sometimes | 521 (35.8%) | 567 (51.3%) | 217 (41.5%) | 1305 (42.3%) |
| Often | 69 (4.7%) | 166 (15.0%) | 162 (31.0%) | 397 (12.9%) |
| **Obesity** |  |  |  |  |
| Never | 1090 (75.1%) | 650 (58.9%) | 292 (55.9%) | 2032 (66.0%) |
| Sometimes | 289 (19.9%) | 336 (30.4%) | 139 (26.6%) | 764 (24.8%) |
| Often | 73 (5.0%) | 118 (10.7%) | 91 (17.4%) | 282 (9.2%) |
| **Damaging properties** |  |  |  |  |
| Never | 1383 (95.0%) | 916 (83.0%) | 337 (64.3%) | 2636 (85.5%) |
| Sometimes | 69 (4.7%) | 168 (15.2%) | 144 (27.5%) | 381 (12.4%) |
| Often | 4 (0.3%) | 19 (1.7%) | 43 (8.2%) | 66 (2.1%) |
| **Running away from home** |  |  |  |  |
| Never | 1450 (99.5%) | 1080 (97.7%) | 474 (90.8%) | 3004 (97.4%) |
| Sometimes | 6 (0.4%) | 19 (1.7%) | 40 (7.7%) | 65 (2.1%) |
| Often | 1 (0.1%) | 6 (0.5%) | 8 (1.5%) | 15 (0.5%) |
| **Skipping school/absenteeism** |  |  |  |  |
| Never | 1439 (98.8%) | 1069 (96.8%) | 490 (93.7%) | 2998 (97.2%) |
| Sometimes | 17 (1.2%) | 32 (2.9%) | 28 (5.4%) | 77 (2.5%) |
| Often | 0 (0.0%) | 3 (0.3%) | 5 (1.0%) | 8 (0.3%) |
| **Social media use** |  |  |  |  |
| 0h | 46 (3.2%) | 29 (2.6%) | 12 (2.3%) | 87 (2.8%) |
| 0-3h | 333 (22.9%) | 208 (18.8%) | 66 (12.7%) | 607 (19.7%) |
| 3-7h | 364 (25.0%) | 257 (23.3%) | 117 (22.5%) | 738 (24.0%) |
| 7-14h | 297 (20.4%) | 225 (20.4%) | 94 (18.0%) | 616 (20.0%) |
| 14-21h | 185 (12.7%) | 128 (11.6%) | 70 (13.4%) | 383 (12.4%) |
| 21-28h | 97 (6.7%) | 88 (8.0%) | 43 (8.3%) | 228 (7.4%) |
| 28-35h | 45 (3.1%) | 56 (5.1%) | 34 (6.5%) | 135 (4.4%) |
| >35h | 89 (6.1%) | 113 (10.2%) | 85 (16.3%) | 287 (9.3%) |
| **Internet gaming use** |  |  |  |  |
| 0h | 165 (11.4%) | 110 (10.0%) | 44 (8.4%) | 319 (10.4%) |
| 0-3h | 377 (26.0%) | 261 (23.7%) | 106 (20.3%) | 744 (24.2%) |
| 3-7h | 325 (22.4%) | 242 (22.0%) | 105 (20.1%) | 672 (21.9%) |
| 7-14h | 230 (15.9%) | 169 (15.3%) | 81 (15.5%) | 480 (15.6%) |
| 14-21h | 151 (10.4%) | 116 (10.5%) | 65 (12.4%) | 332 (10.8%) |
| 21-28h | 73 (5.0%) | 69 (6.3%) | 38 (7.3%) | 180 (5.9%) |
| 28-35h | 41 (2.8%) | 44 (4.0%) | 22 (4.2%) | 107 (3.5%) |
| >35h | 89 (6.1%) | 89 (8.1%) | 61 (11.7%) | 239 (7.8%) |
| **Suicidal ideation** |  |  |  |  |
| Not at all | 1300 (89.5%) | 668 (60.5%) | 194 (37.1%) | 2162 (70.2%) |
| Several days | 129 (8.9%) | 315 (28.5%) | 162 (31.0%) | 606 (19.7%) |
| More than half of the days | 21 (1.4%) | 97 (8.8%) | 111 (21.2%) | 229 (7.4%) |
| Nearly every day | 2 (0.1%) | 25 (2.3%) | 56 (10.7%) | 83 (2.7%) |

Supplementary Table 3 Associations between cluster membership and behavioral, Internet use, and mental health problems using multinominal logistic regression analysis

| **Alcohol or substance abuse** |  | AOR (95% CI) | P-value |
| --- | --- | --- | --- |
| Sometimes vs. None | Cluster 1 (ref) | 1.00 |  |
|  | Cluster 2 | 3.14 (1.93, 5.12)*** | <.001 |
|  | Cluster 3 | 6.14 (3.69, 10.22)*** | <.001 |
| Often vs. None | Cluster 1 (ref) | 1.00 |  |
|  | Cluster 2 | 7.86 (.94, 65.69) | .057 |
|  | Cluster 3 | 27.36 (3.42, 218.68)** | .002 |
| **Tobacco use** |  | AOR (95% CI) | P-value |
| Sometimes vs. None | Cluster 1 (ref) | 1.00 |  |
|  | Cluster 2 | 1.86 (.92, 3.79) | .085 |
|  | Cluster 3 | 4.25 (2.08, 8.70)*** | <.001 |
| Often vs. None | Cluster 1 (ref) | 1.00 |  |
|  | Cluster 2 | 3.31 (.64, 17.28) | .155 |
|  | Cluster 3 | 11.16 (2.32, 53.73)** | .003 |
| **Sleep disturbance** |  | AOR (95% CI) | P-value |
| Sometimes vs. None | Cluster 1 (ref) | 1.00 |  |
|  | Cluster 2 | 2.32 (1.94, 2.77)*** | <.001 |
|  | Cluster 3 | 2.45 (1.91, 3.14)*** | <.001 |
| Often vs. None | Cluster 1 (ref) | 1.00 |  |
|  | Cluster 2 | 5.17 (3.75, 7.12)*** | <.001 |
|  | Cluster 3 | 14.32 (10.09, 20.34)*** | <.001 |
| **Obesity** |  | AOR (95% CI) | P-value |
| Sometimes vs. None | Cluster 1 (ref) | 1.00 |  |
|  | Cluster 2 | 1.91 (1.57, 2.32)*** | <.001 |
|  | Cluster 3 | 1.78 (1.38, 2.30)*** | <.001 |
| Often vs. None | Cluster 1 (ref) | 1.00 |  |
|  | Cluster 2 | 2.76 (2.00, 3.81)*** | <.001 |
|  | Cluster 3 | 4.90 (3.45, 6.96)*** | <.001 |
| **Damaging properties** |  | AOR (95% CI) | P-value |
| Sometimes vs. None | Cluster 1 (ref) | 1.00 |  |
|  | Cluster 2 | 4.00 (2.93, 5.46)*** | <.001 |
|  | Cluster 3 | 9.31 (6.68, 12.96)*** | <.001 |
| Often vs. None | Cluster 1 (ref) | 1.00 |  |
|  | Cluster 2 | 6.34 (2.13, 18.87)** | .001 |
|  | Cluster 3 | 37.63 (13.29, 106.58)*** | <.001 |
| **Running away from home** |  | AOR (95% CI) | P-value |
| Sometimes vs. None | Cluster 1 (ref) | 1.00 |  |
|  | Cluster 2 | 3.91 (1.54, 9.91)** | .004 |
|  | Cluster 3 | 17.33 (7.22, 41.60)*** | <.001 |
| Often vs. None | Cluster 1 (ref) | 1.00 |  |
|  | Cluster 2 | 7.81 (.93, 65.41) | .058 |
|  | Cluster 3 | 18.85 (2.28, 155.66)** | .006 |
| **Skipping school/absenteeism** |  | AOR (95% CI) | P-value |
| Sometimes vs. None | Cluster 1 (ref) | 1.00 |  |
|  | Cluster 2 | 2.63 (1.40, 4.92)** | .003 |
|  | Cluster 3 | 4.24 (2.17, 8.27)*** | <.001 |
| Often vs. None | Cluster 1 (ref) | 1.00 |  |
|  | Cluster 2 | NA† | -- |
|  | Cluster 3 | NA† | -- |
| **Social media usage (per week)** |  | AOR (95% CI) | P-value |
| >0-3h vs. 0h | Cluster 1 (ref) | 1.00 |  |
|  | Cluster 2 | 0.90 (.54, 1.51) | .696 |
|  | Cluster 3 | 0.77 (.37, 1.59) | .481 |
| >3-7h vs. 0h | Cluster 1 (ref) | 1.00 |  |
|  | Cluster 2 | 0.86 (.52, 1.44) | .568 |
|  | Cluster 3 | 1.17 (.58, 2.38) | .663 |
| >7-14h vs. 0h | Cluster 1 (ref) | 1.00 |  |
|  | Cluster 2 | 0.94 (.56, 1.58) | .842 |
|  | Cluster 3 | 1.21 (.59, 2.50) | .598 |
| >14-21h vs. 0h | Cluster 1 (ref) | 1.00 |  |
|  | Cluster 2 | 0.90 (.53, 1.54) | .702 |
|  | Cluster 3 | 1.48 (.71, 3.09) | .295 |
| >21-28h vs. 0h | Cluster 1 (ref) | 1.00 |  |
|  | Cluster 2 | 1.09 (.61, 1.92) | .780 |
|  | Cluster 3 | 1.72 (.79, 3.73) | .172 |
| >28-35h vs. 0h | Cluster 1 (ref) | 1.00 |  |
|  | Cluster 2 | 1.28 (.68, 2.42) | .449 |
|  | Cluster 3 | 2.75 (1.21, 6.25)* | .015 |
| >35h vs. 0h | Cluster 1 (ref) | 1.00 |  |
|  | Cluster 2 | 1.62 (.921, 2.84) | .094 |
|  | Cluster 3 | 3.36 (1.59, 7.08)** | .001 |
| **Internet gaming usage (per week)** |  | AOR (95% CI) | P-value |
| >0-3h vs. 0h | Cluster 1 (ref) | 1.00 |  |
|  | Cluster 2 | 1.14 (.84, 1.55) | .407 |
|  | Cluster 3 | 1.19 (.77, 1.83) | .437 |
| >3-7h vs. 0h | Cluster 1 (ref) | 1.00 |  |
|  | Cluster 2 | 1.25 (.91, 1.71) | .175 |
|  | Cluster 3 | 1.40 (.90, 2.16) | .136 |
| >7-14h vs. 0h | Cluster 1 (ref) | 1.00 |  |
|  | Cluster 2 | 1.27 (.91, 1.79) | .162 |
|  | Cluster 3 | 1.66 (1.05, 2.63)* | .030 |
| >14-21h vs. 0h | Cluster 1 (ref) | 1.00 |  |
|  | Cluster 2 | 1.31 (.91, 1.90) | .153 |
|  | Cluster 3 | 2.06 (1.27, 3.35)** | .003 |
| >21-28h vs. 0h | Cluster 1 (ref) | 1.00 |  |
|  | Cluster 2 | 1.62 (1.05, 2.51)* | .031 |
|  | Cluster 3 | 2.46 (1.41, 4.30)** | .002 |
| >28-35h vs. 0h | Cluster 1 (ref) | 1.00 |  |
|  | Cluster 2 | 1.88 (1.11, 3.18)* | .020 |
|  | Cluster 3 | 2.35 (1.20, 4.60)* | .013 |
| >35h vs. 0h | Cluster 1 (ref) | 1.00 |  |
|  | Cluster 2 | 1.68 (1.11, 2.53)* | .014 |
|  | Cluster 3 | 2.85 (1.70, 4.79)*** | <.001 |
| **Suicide ideation** |  | AOR (95% CI) | P-value |
| Several days vs. Not at all | Cluster 1 (ref) | 1.00 |  |
|  | Cluster 2 | 5.04 (3.97, 6.39)*** | <.001 |
|  | Cluster 3 | 8.60 (6.41,11.54)*** | <.001 |
| More than half of the days vs. Not at all | Cluster 1 (ref) | 1.00 |  |
|  | Cluster 2 | 8.60 (5.21, 14.18)*** | <.001 |
|  | Cluster 3 | 36.44 (21.88, 60.70)*** | <.001 |
| Nearly every day vs. Not at all | Cluster 1 (ref) | 1.00 |  |
|  | Cluster 2 | 22.26 (5.22, 94.87)*** | <.001 |
|  | Cluster 3 | 176.73 (42.55, 734.15)*** | <.001 |

Note: *p<.05, **p<.01, ***p<.001.

†NA: not applicable. The odds ratios were not computed as the cell in the crosstab table had a value of less than 5.
